# Association of Premenopausal Bilateral Oophorectomy With Cognitive Performance and Risk of Mild Cognitive Impairment

**DOI:** 10.1001/jamanetworkopen.2021.31448

**Published:** 2021-11-11

**Authors:** Walter A. Rocca, Christine M. Lohse, Carin Y. Smith, Julie A. Fields, Mary M. Machulda, Michelle M. Mielke

**Affiliations:** 1 Division of Epidemiology, Department of Quantitative Health Sciences, Mayo Clinic, Rochester, Minnesota; 2 Department of Neurology, Mayo Clinic, Rochester, Minnesota; 3 Mayo Clinic Specialized Center of Research Excellence on Sex Differences, Mayo Clinic, Rochester, Minnesota; 4 Division of Clinical Trials and Biostatistics, Department of Quantitative Health Sciences, Mayo Clinic, Rochester, Minnesota; 5 Department of Psychiatry and Psychology, Mayo Clinic, Rochester, Minnesota

## Abstract

**Importance:**

The associations of bilateral oophorectomy among premenopausal women, age at oophorectomy, and use of estrogen therapy after oophorectomy with cognitive performance later in life remain controversial.

**Objective:**

To investigate whether women who underwent premenopausal bilateral oophorectomy were at increased risk of mild cognitive impairment (MCI) and experienced decreased global or domain-specific cognitive performance.

**Design, Setting, and Participants:**

This case-control study and cross-sectional study were made possible by combining data from the Mayo Clinic Study of Aging (MCSA) and the Rochester Epidemiology Project (REP) medical record–linkage system. The studies were conducted among a population-based sample in Olmsted County, Minnesota, consisting of 2732 women aged 50 to 89 years who participated in the MCSA study from 2004 to 2019 and underwent a clinical evaluation and comprehensive cognitive testing. Data were analyzed from January to May 2021.

**Exposures:**

Medical record documentation of bilateral oophorectomy abstracted from a medical record–linkage system (ie, REP).

**Main Outcomes and Measures:**

Odds of MCI and global or domain-specific *z* scores on cognitive tests were measured at the first MCSA visit. The median (IQR) lag time between bilateral oophorectomy performed before menopause and before age 50 years and cognitive evaluation was 30 (22-38) years.

**Results:**

Among 2732 women aged 50 to 89 years (median [IQR] age at evaluation, 74 [66-81] years) who participated in the MCSA, the case-control study included 283 women with MCI (10.4%) and 2449 women without cognitive impairment (89.6%). Bilateral oophorectomy before menopause and before age 46 years was associated with clinically diagnosed MCI (adjusted odds ratio [aOR], 2.21; 95% CI, 1.41-3.45; *P* < .001) compared with no bilateral oophorectomy. The presence of an association with MCI varied by surgical indication, with an association among 259 women with bilateral oophorectomy before menopause and before age 50 years for the indication of benign ovarian condition (aOR, 2.43; 95% CI, 1.36-4.33; *P* = .003) but not for cancer or no ovarian condition. The presence of an association did not vary by estrogen therapy after bilateral oophorectomy, with associations among women aged less than 46 years with estrogen therapy (aOR, 2.56; 95% CI, 1.24-5.31; *P* = .01) and without estrogen therapy (aOR, 2.05; 95% CI, 1.18-3.52; *P* = .01). The cross-sectional study included 625 women with a history of bilateral oophorectomy (median [IQR] age, 75 [70-82] years) and 2107 women without a history of bilateral oophorectomy (median [IQR] age, 73 [65-80] years). Premenopausal bilateral oophorectomy was performed before age 46 years among 161 women and was associated with decreased global cognition *z* score (β, −0.17; 95% CI, −0.32 to −0.03; *P* = .02), attention and executive domain *z* score (β, −0.21; 95% CI, −0.36 to −0.05; *P* = .009), and Short Test of Mental Status score (β, −0.51; 95% CI, −0.95 to −0.08; *P* = .02) compared with no bilateral oophorectomy.

**Conclusions and Relevance:**

This study found that women who underwent bilateral oophorectomy before menopause had increased odds of MCI and poorer performance on cognitive tests approximately 30 years later compared with women who did not undergo bilateral oophorectomy.

## Key Points

**Question:**

Is bilateral oophorectomy performed before spontaneous menopause associated with later-life cognition?

**Findings:**

In a case-control study of 2732 women aged 50 to 89 years who participated in the Mayo Clinic Study of Aging, women who underwent premenopausal bilateral oophorectomy before age 46 years had increased odds of mild cognitive impairment (MCI). In a cross-sectional study in the same group, undergoing premenopausal bilateral oophorectomy before age 46 years was associated with decreased scores on a global cognitive measure, an attention and executive domain measure, and a cognitive screening measure compared with not undergoing bilateral oophorectomy.

**Meaning:**

This study found that bilateral oophorectomy performed before spontaneous menopause and before age 46 years was associated with increased odds of MCI and decreased cognitive performance approximately 30 years later.

## Teaser

This case-control and cross-sectional study investigates the association of premenopausal bilateral oophorectomy with mild cognitive impairment and cognitive test scores in later life.

## Introduction

The hypothesis that surgical removal of the ovaries among premenopausal women may be associated with brain aging outcomes has been under study for more than a decade.^1^ In 2007, we initially reported an association between bilateral oophorectomy performed among women before age 49 years and an increased risk of cognitive impairment or dementia. The risk increased with younger age at oophorectomy and decreased with use of estrogen therapy (ET) after the surgical procedure.^1,2,3^ In 2010, a second study^4^ also found such an association using the linkage of national registries from Denmark. In 2014, a US study^5^ and a French study^6^ additionally found this association. The US and French studies also found decreased risk with ET, thus suggesting that the association between removal of the ovaries and cognitive outcomes is mediated by the abrupt and prolonged estrogen deprivation associated with the surgical procedure.^5,6^ In 2016, we again found the association in a new cohort study among the same Olmsted County population (independent replication in the same population).^7^

In 2019, approximately 12 years after the initial report, a systematic review and meta-analysis was published.^8^ Although 11 studies were considered of adequate quality, 2 studies focused on the association of bilateral oophorectomy before age 46 years with risk of dementia. The meta-analysis found a 70% increased risk of dementia. Two additional studies were published on the same association after 2019.^9,10^ Nevertheless, the association between bilateral oophorectomy and cognitive decline or dementia is not considered established and is not included in mainstream reviews or summaries of the literature on risk factors associated with dementia.^11,12,13^ The major reasons for this lack of recognition are the association of age at which the bilateral oophorectomy is performed with cognitive decline or dementia and controversial data about the association of ET after oophorectomy with cognitive decline or dementia.^2,14^

In this study, we addressed some limitations of previous studies by focusing on premature or early bilateral oophorectomy, focusing on mild cognitive impairment (MCI), and using a comprehensive cognitive battery (9 cognitive tests covering 4 domains). The study was made possible by combining the research infrastructure of the Mayo Clinic Study of Aging (MCSA) and the Rochester Epidemiology Project (REP) medical record–linkage system.

## Methods

This case-control and cross-sectional study follows the Strengthening the Reporting of Observational Studies in Epidemiology (STROBE) reporting guideline. All research activities were approved by the Mayo Clinic and the Olmsted Medical Center institutional review boards. Written informed consent was obtained from all participants.

### Methods of the MCSA

The MCSA is a prospective population-based study of the prevalence and incidence of cognitive decline and MCI and of risk factors associated with them among a representative sample of individuals living in Olmsted County, Minnesota.^15^ In 2004, Olmsted County residents ages 70 to 89 years were enumerated using the REP medical record–linkage system in a random sampling design stratified by age and sex.^15^ In 2012, the study was extended to include individuals aged 50 years and older. The recruitment and follow-up of study participants is ongoing as of August 2021. Additional details about the REP were reported elsewhere.^16,17^

MCSA visits include an interview by a study coordinator, a physician examination, and neuropsychological testing, as described elsewhere.^15,18^ Clinical follow-up visits occur approximately every 15 months. Cognitive evaluation includes 9 tests covering 4 cognitive domains. All tests were administered face to face by a psychometrist under the supervision of a neuropsychologist (including J.A.F. and M.M.M.). The 3 memory tests are the Auditory Verbal Learning Test (AVLT; range, 0%-100% retention)^19^ and the Logical Memory subtest (range, 0%-100% retention) and Visual Reproduction subtest (range, 0%-100% retention) of the Wechsler Memory Scale-Revised.^20^ The 2 attention and executive tests are the Trail Making Test (range, 0-300)^21,22^ and the Digit Symbol subtest (range, 0-93) of the Wechsler Adult Intelligence Scale-Revised (WAIS-R).^23^ The 2 visuospatial tests are the Picture Completion subtest (range, 0-20) and Block Design subtest (range, 0-51) of the WAIS-R.^23^ The 2 language tests are the Boston Naming Test (range, 0-60)^24^ and the Category Fluency Test (range, unlimited).^25^

Using the mean (SD) among 2449 women included in the study who were cognitively unimpaired, test scores were converted to *z* scores, and the mean was found for tests within each domain to obtain domain *z* scores. A global *z* score was calculated by finding the mean of 4 cognitive domain scores. Further details regarding the transformation of raw scores are reported in eTable 1 in the Supplement. In addition, participants were administered a brief cognitive screening measure, the Short Test of Mental Status (range, 0-38).^26^ In this study, we considered the first MCSA visit for each woman (ie, the index date).

### Assessment of MCI and Dementia

Clinical diagnoses of MCI and dementia were determined by a consensus committee including the physician, study coordinator, and neuropsychologist who interpreted cognitive data for each participant. Raw scores of cognitive tests were age adjusted and scaled using normative data from the Mayo Older Americans Normative Studies.^27^ The operational definition of MCI was based on clinical judgment and included a history from the participant and an informant (eg, a family member caring for the individual) and the result of cognitive testing. Published criteria were used for the clinical diagnosis of MCI as follows: cognitive complaint, cognitive function outside of reference range for age, functional activities essentially within the reference range, and no dementia.^28^ A final decision was made after considering education, occupation, and visual or hearing deficits and after reviewing all other participant information. The diagnosis of dementia was based on published criteria.^29^ Participants who performed in the reference range and did not meet the functional criteria for MCI or dementia were considered cognitively unimpaired.

### Assessment of Bilateral Oophorectomy

Details about age at bilateral oophorectomy, surgical indication, and use of ET after oophorectomy were abstracted from medical records by specifically trained abstractors. Oophorectomy was considered to have a benign indication if it was performed for a presumed nonmalignant ovarian condition, such as adnexal mass, cyst, or endometriosis. We used the term no ovarian condition if the bilateral oophorectomy was performed at the time of hysterectomy in the absence of any ovarian condition. Hysterectomy was performed most commonly for uterine fibroids, heavy menstrual bleeding, or chronic pelvic pain. In a minority of women (64 of 603 women with indication data [10.6%]), the indication was primary or metastatic ovarian cancer or the ovaries were removed for the treatment of breast or endometrial cancer.

### Assessment of Other Variables

Demographic characteristics (ie, age, years of education, and self-reported race and ethnicity) were ascertained at the time of the in-clinic visit. Race and ethnicity data were collected because these factors may be modifiers of the associations investigated in this study. However, most women in our study were White and were not Hispanic. Women’s height (in centimeters) and weight (in kilograms) were measured during the in-clinic visit and were used to calculate body mass index (BMI; calculated as weight in kilograms divided by height in meters squared). Depressive symptoms were assessed using the Beck Depression Inventory-II (range, 0-63)^30^; participants with a score of 13 or greater were considered to have depression. Anxiety symptoms were assessed using the Beck Anxiety Inventory (range, 0-63).^31^ The 2 tests were administered face to face by a psychometrist under the supervision of a neuropsychologist (including J.A.F. and M.M.M.). Smoking history (ie, never, current, or former) was obtained by self-report. Medical conditions present at first MCSA visit were determined for each participant by abstraction of the medical record from the REP medical record–linkage system.^15,16,17^ Apolipoprotein E (*APOE*) ε4 genotyping was performed from a blood sample obtained during the in-clinic visit.

### Statistical Analysis

Case-control analyses were performed to compare women with MCI (ie, the case group) and women without MCI (ie, the control group) at the time of first MCSA visit (ie, index date). Odds ratios (ORs) and 95% CIs were calculated using logistic regression models adjusted initially for age at the time of cognitive evaluation (continuous variable). In additional models, we adjusted for age at the time of cognitive evaluation (continuous variable), years of education (≤12 vs 13-16 vs >16 years), and *APOE* genotype (ie, with ε4 variant vs all others), finding adjusted odds ratios (aORs). Case-control analyses were conducted overall and stratified by age at bilateral oophorectomy, use of ET, surgical indication, and lag time between bilateral oophorectomy and time of cognitive evaluation.

In addition, cross-sectional analyses were performed to compare cognitive performance among women with or without bilateral oophorectomy at the time of their first MCSA visit (ie, index date) using linear regression models adjusted for age at time of cognitive evaluation (continuous variable), years of education (≤12 vs 13-16 vs >16 years), and *APOE* genotype (ie, with ε4 variant vs all others). We compared women with bilateral oophorectomy before menopause at ages less than 46 years and at ages 46 to 49 years vs women without bilateral oophorectomy.

Statistical analyses were performed using SAS statistical software version 9.4 (SAS Institute) and R statistical software version 3.6.2 (R Project for Statistical Computing). Tests of statistical significance were conducted at the 2-tailed α level of .05. Data were analyzed from January to May 2021.

## Results

### Characteristics at Index Date

**Figure 1.**
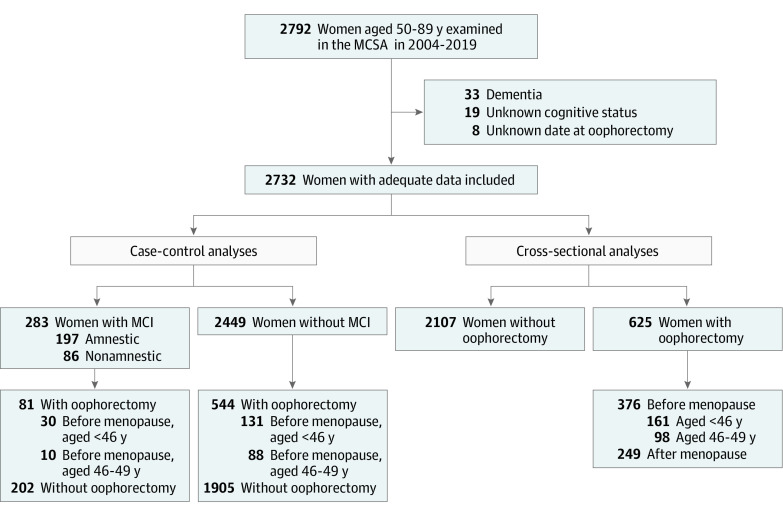
Flowchart of Case-Control and Cross-Sectional Analyses The case-control analyses compared the frequency of history of bilateral oophorectomy among women with or without mild cognitive impairment (MCI). The cross-sectional analyses compared the cognitive performance among women with a history of bilateral oophorectomy before menopause and at age less than 46 years or at ages 46 to 49 years with that among women without a history of bilateral oophorectomy. MCSA indicates Mayo Clinic Study of Aging.

A total of 2792 women were aged 50 to 89 years at their first MCSA visit from 2004 to 2019 and were considered for inclusion. However, 33 women with a diagnosis of dementia and 19 women with unknown cognitive status at the time of cognitive evaluation were excluded. In addition, we excluded 8 women with an unknown date of bilateral oophorectomy, leaving 2732 women included in the analyses (median [IQR] age at evaluation, 74 [66-81] years). Among 2722 women with race data, there were 22 Asian women (0.8%), 9 Black women (0.3%), 2679 White women (98.4%), and 12 women with other race (0.4%), and among 2718 women with ethnicity data, there were 14 Hispanic women (0.5%). Among the study population, 625 women (22.9%) had a history of bilateral oophorectomy and 2107 women (77.1%) did not (Figure 1).

A comparison of demographic and clinical characteristics of women with and without bilateral oophorectomy at the time of cognitive evaluation (ie, the index date) is shown in Table 1. Women with a history of bilateral oophorectomy at any age were older (median [IQR] age at evaluation, 75 [70-82] years vs 73 [65-80] years; *P* < .001) and had more comorbid conditions, including hypertension (461 women [73.8%] vs 1345 women [63.8%]; *P* < .001), diabetes (111 women [17.8%] vs 291 women [13.8%]; *P* = .01), heart disease (including atrial fibrillation, coronary artery disease, and congestive heart failure; 194 women [31.0%] vs 540 women [25.6%]; *P* = .007), and stroke (31 women [5.0%] vs 67 women [3.2%]; *P* = .04). Table 1 also provides details about bilateral oophorectomy for all women and for women who underwent bilateral oophorectomy before menopause and before age 50 years. Of women who underwent bilateral oophorectomy, 376 women (60.2%) had oophorectomy before menopause and 249 women (39.8%) had the procedure after menopause. Oophorectomy was before menopause and before age 50 years among 259 women (41.4%) (Figure 1). Among these women, the median (IQR) time between the date of bilateral oophorectomy and the date of cognitive evaluation was 30 (22-38) years. Of 161 women who underwent bilateral oophorectomy before menopause and before age 46 years, 95 women (59.0%) did not receive ET and 46 women (28.6%) received oral conjugated equine estrogen (unopposed).

**Table 1.** Demographic and Clinical Characteristics

Characteristic	Women, No. (%) (N = 2732)		*P* value
	With bilateral oophorectomy (n = 625)	Without bilateral oophorectomy (n = 2107)	
Age at evaluation, median (IQR), y	75 (70-82)	73 (65-80)	<.001
Race^a^			
Asian	3 (0.5)	19 (0.9)	.61
Black	2 (0.3)	7 (0.3)	
White	614 (98.6)	2065 (98.4)	
Other	4 (0.6)	8 (0.4)	
Hispanic ethnicity^a^	3 (0.5)	11 (0.5)	.90
Years of education^b^			
<9	13 (2.1)	44 (2.1)	.30
9-12	216 (34.6)	691 (32.8)	
13-16	308 (49.4)	1042 (49.5)	
>16	87 (13.9)	327 (15.5)	
BMI^c^			
<25	187 (30.3)	679 (33.0)	.10
25.0-29.9	212 (34.4)	719 (35.0)	
≥30	218 (35.3)	658 (32.0)	
BDI^d^			
<13	566 (92.5)	1911 (92.4)	.98
≥13	46 (7.5)	156 (7.6)	
BAI, median (IQR)^e^	2 (0-5)	2 (0-5)	.35
Chronic condition			
Hypertension	461 (73.8)	1345 (63.8)	<.001
Dyslipidemia	491 (78.6)	1583 (75.1)	.08
Diabetes	111 (17.8)	291 (13.8)	.01
Heart disease	194 (31.0)	540 (25.6)	.007
Stroke	31 (5.0)	67 (3.2)	.04
Smoking status^f^			
Never	412 (65.9)	1304 (62.0)	.07
Current or former	213 (34.1)	801 (38.0)	
*APOE* genotype^g^			
With ε4 variant	162 (26.7)	543 (27.4)	.73
Other	444 (73.3)	1435 (72.6)	
**625 women with bilateral oophorectomy^h^**			
Age at bilateral oophorectomy before menopause, y			
Median (IQR)	47 (43-51)	NA	NA
<40	51 (8.2)	NA	
40-45	110 (17.6)	NA	
46-49	98 (15.7)	NA	
≥50	117 (18.7)	NA	
Age at bilateral oophorectomy after menopause, y			
Median (IQR)	63 (58-70)	NA	NA
<60	86 (13.8)	NA	
≥60	163 (26.1)	NA	
Indication for bilateral oophorectomy^i^			
Cancer	64 (10.6)	NA	NA
Benign ovarian condition	156 (25.9)	NA	
No ovarian condition	383 (63.5)	NA	
Hysterectomy status			
None	29 (4.6)	NA	NA
Previous	56 (9.0)	NA	
Concurrent	540 (86.4)	NA	
Lag time from bilateral oophorectomy to cognitive evaluation, y^j^			
Median (IQR)	19 (10-29)	NA	NA
<20	334 (53.4)	NA	
≥20	291 (46.6)	NA	
**259 women with bilateral oophorectomy before menopause and age <50 y^h^**			
Age at bilateral oophorectomy, y			
Median (IQR)	44 (41-47)	NA	NA
<40	51 (19.7)	NA	
40-45	110 (42.5)	NA	
46-49	98 (37.8)	NA	
Indication for bilateral oophorectomy^k^			
Cancer	17 (7.0)	NA	NA
Benign ovarian condition	77 (32.0)	NA	
No ovarian condition	147 (61.0)	NA	
Hysterectomy status			
None	3 (1.2)	NA	NA
Before	11 (4.2)	NA	
Concurrent	245 (94.6)	NA	
Lag time from bilateral oophorectomy to cognitive evaluation, y^j^			
Median (IQR)	30 (22-38)	NA	NA
<30	132 (51.0)	NA	
≥30	127 (49.0)	NA	

Abbreviations: *APOE*, apolipoprotein E; BAI, Beck Anxiety Inventory; BDI, Beck Depression Inventory II; BMI, body mass index (calculated as weight in kilograms divided by height in meters squared); IQR, interquartile range; NA, not applicable.

^a^ Race data were missing for 2 women with and 8 women without bilateral oophorectomy; ethnicity data were missing for 4 women with and 10 women without bilateral oophorectomy.

^b^ Education data were missing for 1 woman with and 3 women without bilateral oophorectomy.

^c^ BMI data were missing for 8 women with and 51 women without bilateral oophorectomy.

^d^ BDI data were missing for 13 women with and 40 women without bilateral oophorectomy.

^e^ BAI data were missing for 4 women with and 11 women without bilateral oophorectomy.

^f^ Smoking status data were missing for 2 women without bilateral oophorectomy.

^g^ *APOE* genotype data were missing for 19 women with and 129 women without bilateral oophorectomy.

^h^ Findings for characteristics that do not apply to women without a history of bilateral oophorectomy are indicated with NA.

^i^ Indication data were missing for 22 women.

^j^ Lag time from bilateral oophorectomy to the time of cognitive evaluation was stratified at approximately the median of the distribution.

^k^ Indication data were missing for 18 women.

### Case-Control Study

There were 283 women (10.4%) with MCI, including 197 women with amnestic MCI (69.6%) and 86 women with nonamnestic MCI (30.4%), and 2449 women (89.6%) who were cognitively unimpaired at the time of cognitive evaluation (Figure 1). The results for case-control analyses of the association of bilateral oophorectomy with MCI are summarized in Table 2. Bilateral oophorectomy overall, at age 46-49 years, and at age ≥50 years was not associated with increased odds of MCI. However, the odds of MCI were statistically significantly increased among women who underwent bilateral oophorectomy before age 46 years (aOR, 2.21; 95% CI, 1.41-3.45; *P* < .001) compared with women who did not undergo bilateral oophorectomy. The aOR was statistically significant for amnestic MCI (1.87; 95% CI, 1.07-3.26; *P* = .03) and nonamnestic MCI (2.96; 95% CI, 1.56-5.62; *P* < .001) (Table 2, footnote c).

**Table 2.** Associations of Bilateral Oophorectomy With MCI at Time of Cognitive Evaluation

	Women, No. (%)		OR (95% CI)^a^	*P* value	aOR (95% CI)^b^	*P* value
	MCI (n = 283)	Unimpaired (n = 2449)				
**Bilateral oophorectomy strata**						
Total						
Without bilateral oophorectomy	202 (71.4)	1905 (77.8)	1 [Reference]	NA	1 [Reference]	NA
With bilateral oophorectomy	81 (28.6)	544 (22.2)	1.26 (0.95-1.66)	.11	1.26 (0.94-1.68)	.13
Age at bilateral oophorectomy before menopause, y						
<46^c^	30 (10.6)	131 (5.4)	2.11 (1.37-3.25)	<.001	2.21 (1.41-3.45)	<.001
46-49	10 (3.5)	88 (3.6)	1.01 (0.51-2.00)	.97	0.79 (0.37-1.68)	.54
≥50	14 (5.0)	103 (4.2)	1.20 (0.67-2.16)	.54	1.25 (0.69-2.26)	.47
Age at bilateral oophorectomy after menopause, y						
<60	9 (3.2)	77 (3.1)	1.16 (0.57-2.38)	.68	1.24 (0.60-2.58)	.56
≥60	18 (6.4)	145 (5.9)	0.85 (0.51-1.43)	.54	0.84 (0.49-1.46)	.54
**259 women with bilateral oophorectomy before menopause and age <50 y**						
ET for bilateral oophorectomy at age <46 y^d^^,^^e^						
Without	19 (6.7)	76 (3.1)	1.93 (1.13-3.29)	.02	2.05 (1.18-3.52)	.01
With	11 (3.9)	55 (2.3)	2.49 (1.25-4.94)	.009	2.56 (1.24-5.31)	.01
ET for bilateral oophorectomy at age 46-49 y^e^^,^^f^						
Without	6 (2.1)	47 (1.9)	0.98 (0.41-2.35)	.97	0.91 (0.38-2.20)	.83
With	4 (1.4)	41 (1.7)	1.07 (0.37-3.04)	.90	0.57 (0.13-2.43)	.45
Indication for bilateral oophorectomy^g^						
Cancer	2 (0.7)	15 (0.6)	0.96 (0.21-4.34)	.96	1.01 (0.22-4.64)	.99
Benign ovarian condition	17 (6.1)	60 (2.5)	2.44 (1.38-4.31)	.002	2.43 (1.36-4.33)	.003
No ovarian condition	16 (5.8)	131 (5.4)	1.22 (0.70-2.11)	.48	1.07 (0.58-1.96)	.83
Lag time from bilateral oophorectomy to clinical evaluation, y^h^						
<30	12 (4.2)	120 (4.9)	1.49 (0.80-2.80)	.21	1.24 (0.62-2.47)	.54
≥30	28 (9.9)	99 (4.0)	1.76 (1.12-2.77)	.01	1.81 (1.13-2.89)	.01

Abbreviations: aOR, adjusted odds ratio*; APOE*, apolipoprotein E; ET, estrogen therapy; MCI, mild cognitive impairment; NA, not applicable; OR, odds ratio.

^a^ ORs and CIs were calculated using logistic regression models adjusted for age at the time of cognitive evaluation (continuous variable).

^b^ aORs and CIs were calculated using logistic regression models adjusted for age at the time of cognitive evaluation (continuous variable), years of education (ie, ≤12 vs 13-16 vs >16 y), and *APOE* genotype (ie, vs ε4 variant vs other).

^c^ The aOR for amnestic MCI was 1.87 (95% CI, 1.07-3.26; *P* = .03). The aOR for nonamnestic MCI was 2.96 (95% CI, 1.56-5.62; *P* < .001). We conducted an additional mediation analysis including hypertension, dyslipidemia, diabetes, heart disease, and stroke in the model. The aOR was 2.07 (95% CI, 1.32-3.26; *P* = .002).

^d^ Among women with bilateral oophorectomy before menopause and at age less than 46 years, the aORs of MCI were not statistically significantly different between women with and without ET (*P* = .62).

^e^ Women were considered with ET if they received ET after bilateral oophorectomy through age 50 years or longer and without ET if they did not receive ET or stopped before age 50 years (approximate age at spontaneous menopause).

^f^ Among women with bilateral oophorectomy before menopause and at ages 46 to 49 years, the aORs of MCI were not statistically significantly different between women with and without ET (*P* = .59).

^g^ 18 women with bilateral oophorectomy before menopause and at age less than 50 years who were missing indication data were excluded.

^h^ Lag time from bilateral oophorectomy to the time of cognitive evaluation was stratified at approximately the median of the distribution.

The presence of an association did not vary between women who did or did not receive ET after the bilateral oophorectomy, with associations with MCI in the age less than 46 years strata for women with ET (aOR, 2.56; 95% CI, 1.24-5.31; *P* = .01) and without ET (aOR, 2.05; 95% CI, 1.18-3.52; *P* = .01) and no association in either group in the age 46 to 49 years strata (with ET: aOR, 0.57; 95% CI, 0.13-2.43; *P* = .45; without ET: aOR, 0.91; 95% CI, 0.38-2.20; *P* = .83) compared with women with no bilateral oophorectomy. The aOR was not significantly different for women who did vs did not receive ET after bilateral oophorectomy in the age younger than 46 years (*P* = .62) or age 46 to 49 years (*P* = .59) strata. The odds were statistically significantly increased among women who underwent oophorectomy before age 50 years for a benign ovarian condition (2.43; 95% CI, 1.36-4.33; *P* = .003) but not for women without an ovarian condition or with a cancer indication compared with women with no bilateral oophorectomy. The odds of MCI were statistically significantly increased among women with a longer lag time (ie, ≥30 years) between the surgical procedure before age 50 years and cognitive evaluation (1.81; 95% CI, 1.13-2.89; *P* = .01) but not among women with a shorter lag time (ie, <30 years; 1.24; 95% CI, 0.62-2.47; *P* = .54). However, this difference may be associated with the younger age at the time of the oophorectomy among women with a longer lag time. There was no association with MCI among women who underwent bilateral oophorectomy after menopause before age 60 years or at age 60 years or older (Table 2) vs women without bilateral oophorectomy.

### Cross-Sectional Comparison of Cognitive Performance

**Figure 2.**
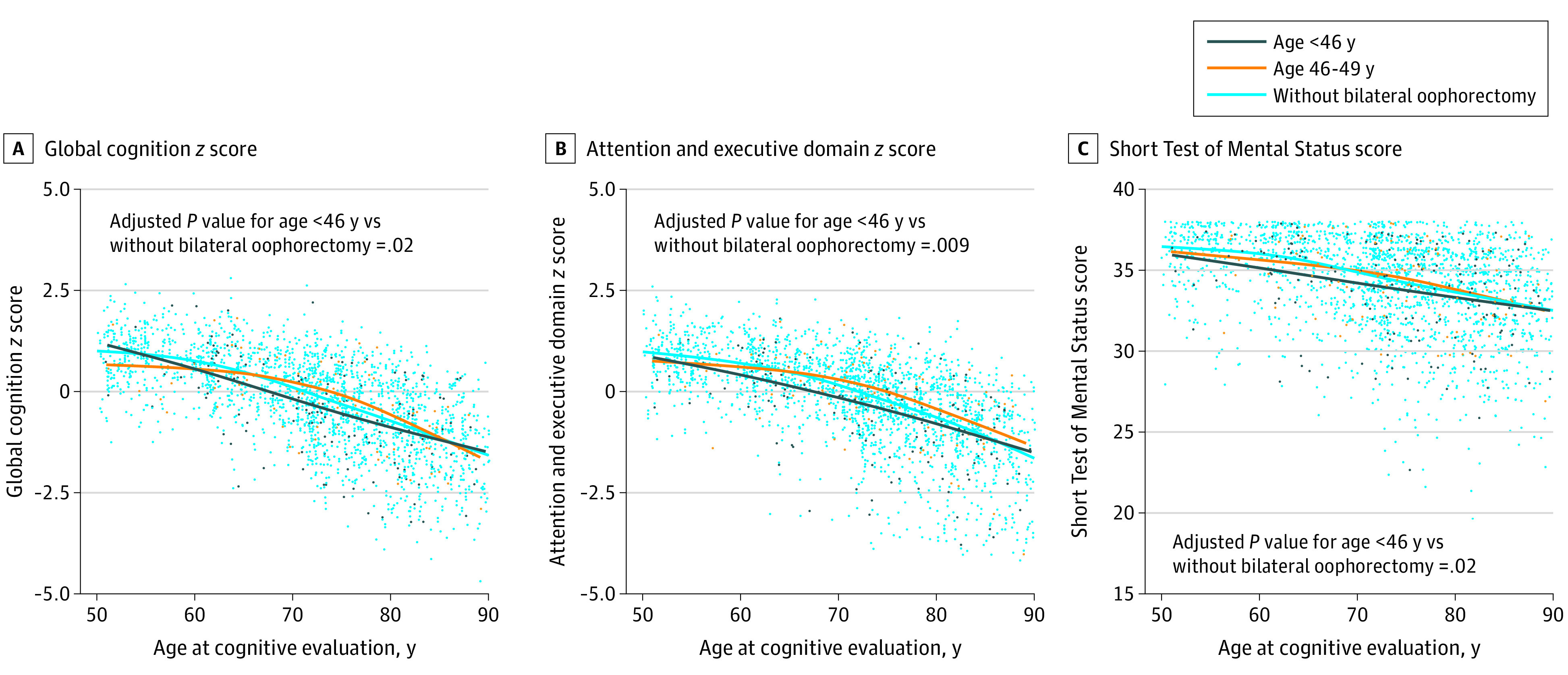
Plots of Cognitive Test Scores by Age Women with bilateral oophorectomy before menopause were stratified by age at the time of oophorectomy as less than 46 years and 46 to 49 years. For the 3 measures shown, women who underwent bilateral oophorectomy before menopause and before age 46 years had statistically significantly decreased scores compared with women who did not undergo bilateral oophorectomy. *P* values were calculated using linear regression models adjusted for age at the time of cognitive evaluation (continuous variable), years of education (ie, ≤12 vs 13-16 vs >16 years), and *APOE* genotype (ie, with ε4 variant vs all others). For the Short Test of Mental Status score, a small amount of random variability was added to each data point (ie, jitter) to better visualize the distribution. Only 1 cognitive assessment (of the full battery of tests) was available for each woman (cross-sectional analyses).

Cognitive performance among women with and without bilateral oophorectomy using a global cognitive score, 4 cognitive domain scores, and the Short Test of Mental Status is shown in eTable 2 in the Supplement. The sample was stratified into 4 strata by 10-year age intervals. Bilateral oophorectomy before age 46 years, compared with no bilateral oophorectomy, was associated with decreased global cognition *z* score (β, −0.17; 95% CI, −0.32 to −0.03; *P* = .02), attention and executive domain *z* score (β, −0.21; 95% CI, −0.36 to −0.05; *P* = .009), and Short Test of Mental Status score (β, −0.51; 95% CI, −0.95 to −0.08; *P* = .02) (eTable 2 in the Supplement; Figure 2). Comparing the 3 curves in the 3 panels of Figure 2, decreased cognitive performance among women who underwent bilateral oophorectomy before age 46 years was most evident from ages 60 to 80 years. Bilateral oophorectomy before age 40 years, which was performed among a smaller group of 51 women, was again associated with decreased global cognition *z* score (β, −0.40; 95% CI, −0.66 to −0.15; *P* = .002), attention and executive domain *z* score (β, −0.38; 95% CI, −0.65 to −0.11; *P* = .005 ), and Short Test of Mental Status score (β, −0.96; 95% CI, −1.71 to −0.21; *P* = .01), as well as visuospatial *z* score (β, −0.30; 95% CI, −0.57 to −0.03; *P* = .03 ) and language *z* score (β, −0.34; 95% CI, −0.61 to −0.06; *P* = .02 ) compared with no bilateral oophorectomy (eTable 2 in the Supplement, footnote d).

## Discussion

### Major Findings

To our knowledge, this case-control and cross-sectional study was the first to report an association between bilateral oophorectomy performed before menopause and before age 46 years and the prevalence of clinically diagnosed MCI. In addition, we found that bilateral oophorectomy performed before menopause and before age 46 years was associated with decreased cognitive performance measured on a battery of 9 cognitive tests grouped into 4 cognitive domains. However, we did not find that the presence of an association varied by use of ET as reported in other studies.^1,2,5,6^

This study has several strengths and unique features. First, women in the study underwent a battery of 9 cognitive tests evaluating performance in 4 cognitive domains. This suggests that our cognitive data were comprehensive. Similarly, the definition of MCI was based on published criteria, and MCI was diagnosed by an experienced group of investigators.^15,28^ The documentation of bilateral oophorectomy was obtained via extensive review of medical records in a record-linkage system. Women were not asked to remember or report past surgical procedures, which suggests that recall bias should be minimized.

Women invited to participate in the MCSA were selected at random from the full enumeration of the population (REP census). Although a segment of invited women did not agree to participate and be tested, our design may have limited selection biases encountered in studies of women recruited from a dementia referral center or from a single medical facility.^15^

### Comparison With Other Studies

A 2019 systematic review and meta-analysis^8^ of the association between bilateral oophorectomy and cognitive decline or dementia identified 2 studies^1,7^ that found a 70% increased risk of dementia restricted to women who were younger than age 46 years at the time of oophorectomy. However, the remaining studies that did not restrict the age at which bilateral oophorectomy was performed found inconsistent results. Two additional studies on the same association were published after 2019.^9,10^ In 2021, a group of investigators from Thailand found results suggesting an association between surgical menopause and decreased frontal lobe function and increased prevalence of MCI. Unfortunately, the investigators did not perform a formal case-control analysis.^32,33^ Our study focused on premature or early bilateral oophorectomy because age at the time of oophorectomy may be biologically important.^14^ Indeed, we did not find an increased risk of MCI among women who underwent premenopausal bilateral oophorectomy at ages 46 to 49 years or 50 years or older. Similarly, we found no increased risk associated with bilateral oophorectomy performed after menopause at any age.

### Interpretation

Based on our own work and on our continuing review of the literature, we have speculated on the possible interpretations of this association between bilateral oophorectomy and cognitive decline or dementia.^1,3,34^ In brief, we can consider 5 possible mechanistic interpretations: (1) The association may be noncausal and may be confounded by genetic variants or other risk factors shared between bilateral oophorectomy and cognitive decline or dementia. (2) The association may be causal and mediated by an abrupt and prolonged decrease in levels of circulating estrogen. (3) The association may be causal and mediated by an abrupt and prolonged decrease in levels of circulating progesterone or testosterone. (4) The association may be causal and mediated by increased release of gonadotropins by the pituitary gland (ie, disruption of the hypothalamus-pituitary-ovarian axis). (5) The association may be causal and mediated by more complex interactions among more than 1 hormonal deprivation (ie, endocrine disruption), genetic variant (eg, *APOE* genotypes), and other risk or protective factors (eg, smoking or obesity).^34^

Interestingly, women who underwent bilateral oophorectomy in our study had an increased frequency of cardiovascular risk factors, heart disease, and stroke at the time of their cognitive evaluation compared with women who did not undergo bilateral oophorectomy. However, in a mediation analysis, we found that after the inclusion of cardiovascular risk factors or events in the regression model the association remained. Therefore, the association may be partly mediated by degenerative brain pathology rather than by cardiovascular pathology. In addition, it is possible that the biological mechanisms are different in different subgroups of women. Additional research is needed to clarify the biological explanation of the association.

### Limitations

This study has several limitations. First, our analyses were either case-control or cross-sectional. Cognitive performance and history of bilateral oophorectomy were assessed at one point in time only among women who agreed to participate in the MCSA study. The median time between the date of bilateral oophorectomy and the date of cognitive evaluation for women with bilateral oophorectomy before age 50 years was 30 years. We do not have information about women who underwent bilateral oophorectomy in the same community and died or moved away before the time of the study, did not get sampled, or did not participate in the study. This study design may be associated with bias because of left censoring and nonparticipation.^15^ In addition, 1 cognitive assessment at a given age was used for each woman. Our findings should be replicated in a longitudinal study with serial cognitive testing over a long follow-up period.

Second, the study included women residing in Olmsted County, Minnesota, and most were White. Thus, results may not be generalizable to other populations with different socioeconomic or racial and ethnic characteristics. Third, because of the retrospective, observational nature of the study, we cannot derive causal inferences. Fourth, the sample size and corresponding statistical power were inadequate for some analyses. For example, we did not have adequate power to compare oral vs transdermal use of ET. In addition, because of the historical time frame of the study, 59.0% of the 161 women who underwent bilateral oophorectomy before menopause and before age 46 years did not receive ET, and 28.6% received oral conjugated equine estrogen (unopposed).

## Conclusions

Our study found that a positive history of premenopausal bilateral oophorectomy performed before age 46 years was associated with MCI. This finding suggests that physicians treating women with premenopausal bilateral oophorectomy need to be aware of their patients’ risk of cognitive impairment or MCI and should consider implementing treatment-monitoring plans. These findings, in conjunction with the results of other studies finding associations of premenopausal bilateral oophorectomy with risk of multiple chronic conditions, may help women at mean risk levels of ovarian cancer to better evaluate the risk to benefit ratio of undergoing bilateral oophorectomy prior to spontaneous menopause for the prevention of ovarian cancer.^7^

## Notes

Supplement. **eTable 1.** Transformation of Raw Scores from 9 Cognitive Tests Into Single Test, Domain, and Global *z* Scores **eTable 2.** Associations of Bilateral Oophorectomy Before Menopause and at Ages <46 and 46-49 y with *z* Score and Short Test of Mental Status Score at Cognitive Evaluation
