# Supplementary material for: Association of Premenopausal Bilateral Oophorectomy With Cognitive Performance and Risk of Mild Cognitive Impairment
Source: JAMA Netw Open. 2021 Nov 11;4(11):e2131448. doi: 10.1001/jamanetworkopen.2021.31448 (PMC8586907; doi:10.1001/jamanetworkopen.2021.31448)
Supplement: Supplement. — eTable 1. Transformation of Raw Scores from 9 Cognitive Tests Into Single Test, Domain, and Global z Scores eTable 2. Associations of Bilateral Oophorectomy Before Menopause and at Ages <46 and 46-49 y with z Score and Short Test of Mental Status Score at Cognitive Evaluation [file jamanetwopen-e2131448-s001.pdf]

Supplemental Online Content

Rocca WA, Lohse CM, Smith CY, Fields JA, Machulda MM, Mielke MM. Association of premenopausal bilateral oophorectomy with cognitive performance and risk of mild cognitive impairment. *JAMA Netw Open*. 2021;4(11):e2131448. doi:10.1001/jamanetworkopen.2021.31448

eTable 1. Transformation of Raw Scores from 9 Cognitive Tests Into Single Test, Domain, and Global *z* Scores

eTable 2. Associations of Bilateral Oophorectomy Before Menopause and at Ages <46 and 46-49 y with *z* Score and Short Test of Mental Status Score at Cognitive Evaluation

This supplemental material has been provided by the authors to give readers additional information about their work.

© 2021 Rocca WA et al. *JAMA Network Open.*

eTable 1. Transformation of Raw Scores from 9 Cognitive Tests Into Single Test, Domain, and Global *z* Scores

Step	Description
1. Select 9 tests[a]	Memory tests (3):<br>• Auditory Verbal Learning Test (AVLT, score range 0%-100% retention)[19]<br>• Logical Memory subtest of the Wechsler Memory Scale-Revised (WMS-R, score range 0%-100% retention)[20]<br>• Visual Reproduction subtest of the Wechsler Memory Scale-Revised (WMS-R, score range 0%-100% retention)[20]<br>Attention/executive tests (2):<br>• Trail Making Test (score range 0-300)[21,22,b]<br>• Digit Symbol subtest of the Wechsler Adult Intelligence Scale-Revised (WAIS-R, score range 0-93)[23]<br>Visuospatial tests (2):<br>• Picture Completion subtest of the Wechsler Adult Intelligence Scale-Revised (WAIS-R, score range 0-20)[23]<br>• Block Design subtest of the Wechsler Adult Intelligence Scale-Revised (WAIS-R, score range 0-51)[23]<br>Language tests (2):<br>• Boston Naming Test (score range 0-60)[24]<br>• Category Fluency Test (score range unlimited)[25]
2. Define a reference population	We used 2449 women who were 50-89 years old at their first contact for the MCSA study from 2004 to 2019, had adequate cognitive data, and were cognitively unimpaired.[c] We derived the following statistical parameters:<br>• Reference mean and SD at the single test level<br>• Reference mean and SD at the domain level<br>• Reference mean and SD at the global level
3. Calculate a single test *z* score	We subtracted the reference mean from the crude score of each person and divided by the refence SD. Due to the *z* scoring, the distribution has mean = 0 and SD = 1.
4. Calculate a domain *z* score	We averaged the *z* score across 3 tests for memory and 2 tests for the remaining domains. We then subtracted the reference mean and divided by the reference SD at the domain level.
5. Calculate a global *z* score	We averaged the 4 domain scores for each person. We then subtracted the reference mean and divided by the reference SD at the global level.

Abbreviations: MCSA, Mayo Clinic Study of Aging; SD, standard deviation

[a] The tests were administered face-to-face by a psychometrist, under the supervision of a neuropsychologist (including J.A.F. and M.M.M.).
[b] The scale for the Trail Making Test was reversed to align the interpretation with the remaining 8 scales (higher scores = better performance).
[c] We required adequate tests to calculate at least 1 of the 4 cognitive domains. For the memory domain, we required at least 2 of 3 tests. For the other 3 domains, we required both of the 2 tests.

© 2021 Rocca WA et al. *JAMA Network Open*.

eTable 2. Associations of Bilateral Oophorectomy Before Menopause and at Ages <46 and 46-49 y with *z* Score and Short Test of Mental Status Score at Cognitive Evaluation

**Score by age at evaluation, y**	**A With bilateral oophorectomy <46 y** (n = 161)[a] Median (IQR)	**B With bilateral oophorectomy 46-49 y** (n = 98)[b] Median (IQR)	**C Without bilateral oophorectomy** (n = 2107)[c] Median (IQR)	**β (95% CI) *P* value[d] A vs. C**	**β (95% CI) *P* value[e] B vs. C**
Global cognition *z* score					
50-59	0.76 (0.39 to 0.99)	0.24 (-0.16 to 0.66)	0.99 (0.48 to 1.39)	-0.17	0.13
60-69	0.72 (-0.19 to 1.13)	0.44 (0.12 to 0.92)	0.53 (-0.03 to 1.01)	(-0.32 to -0.03)	(-0.06 to 0.32)
70-79	-0.43 (-1.08 to 0.15)	0.13 (-0.39 to 0.53)	-0.24 (-0.86 to 0.40)	0.02	0.17
80-89	-1.11 (-1.80 to -0.34)	-1.30 (-1.90 to -0.50)	-0.97 (-1.81 to -0.35)		
Memory *z* score					
50-59	0.85 (0.26 to 1.09)	0.27 (0.11 to 1.07)	0.84 (0.21 to 1.39)	-0.08	0.14
60-69	0.38 (-0.12 to 1.21)	0.55 (-0.26 to 1.15)	0.42 (-0.14 to 1.00)	(-0.24 to 0.08)	(-0.06 to 0.34)
70-79	-0.64 (-1.24 to 0.12)	-0.26 (-0.69 to 0.61)	-0.25 (-1.04 to 0.44)	0.31	0.17
80-89	-0.90 (-1.63 to 0.05)	-1.08 (-1.67 to -0.58)	-0.84 (-1.61 to -0.08)		
Attention/Executive *z* score					
50-59	0.41 (-0.02 to 1.17)	0.80 (0.15 to 0.97)	0.91 (0.45 to 1.29)	-0.21	0.17
60-69	0.44 (-0.03 to 0.92)	0.46 (0.07 to 0.95)	0.57 (0.10 to 0.97)	(-0.36 to -0.05)	(-0.02 to 0.36)
70-79	-0.09 (-0.99 to 0.20)	0.16 (-0.28 to 0.67)	-0.12 (-0.65 to 0.42)	0.009	0.09
80-89	-0.85 (-1.74 to -0.36)	-0.78 (-1.27 to -0.10)	-0.85 (-1.71 to -0.25)		
Visuospatial *z* score					
50-59	1.05 (0.24 to 1.49)	0.13 (-0.35 to 0.35)	0.81 (0.20 to 1.43)	-0.12	-0.03
60-69	0.20 (-0.52 to 1.23)	0.31 (-0.15 to 1.10)	0.35 (-0.29 to 0.94)	(-0.27 to 0.04)	(-0.22 to 0.17)
70-79	-0.48 (-1.35 to 0.26)	0.09 (-0.66 to 0.53)	-0.18 (-0.86 to 0.42)	0.13	0.79
80-89	-0.53 (-1.49 to -0.07)	-0.99 (-1.56 to -0.32)	-0.68 (-1.47 to 0.00)		
Language *z* score					
50-59	0.30 (-0.60 to 1.06)	0.02 (-0.26 to 0.41)	0.62 (0.12 to 1.13)	-0.12	0.15
60-69	0.35 (-0.47 to 1.03)	0.58 (-0.02 to 0.99)	0.40 (-0.13 to 0.96)	(-0.28 to 0.04)	(-0.05 to 0.36)
70-79	-0.03 (-0.72 to 0.53)	0.14 (-0.77 to 0.70)	-0.18 (-0.81 to 0.50)	0.15	0.14
80-89	-0.73 (-1.49 to -0.13)	-0.93 (-1.37 to -0.21)	-0.67 (-1.49 to -0.10)		

© 2021 Rocca WA et al. *JAMA Network Open*.

eTable 2. (continued)

Score by age at evaluation, y	**A With bilateral oophorectomy <46 y** (n = 161)[a] Median (IQR)	**B With bilateral oophorectomy 46-49 y** (n = 98)[b] Median (IQR)	**C Without bilateral oophorectomy** (n = 2107)[c] Median (IQR)	**β (95% CI) *P* value[d] A vs. C**	**β (95% CI) *P* value[e] B vs. C**
Short Test of Mental Status score[f]					
50-59	36 (35 to 37)	36 (36 to 37)	37 (35 to 38)	-0.51	0.24
60-69	36 (34 to 37)	35 (34 to 37)	36 (34 to 37)	(-0.95 to -0.08)	(-0.30 to 0.79)
70-79	34 (31 to 36)	36 (34 to 36)	35 (33 to 36)	0.02	0.38
80-89	33 (31 to 35)	33 (31 to 34)	34 (31 to 35)		

Abbreviations: *APOE*, apolipoprotein E; IQR, interquartile range (25th percentile to, 75th percentile); y, years.

[a] Women with bilateral oophorectomy before menopause and <46 years included 12 age 50-59, 40 age 60-69, 58 age 70-79, and 51 age 80-89 at the time of cognitive evaluation.

[b] Women with bilateral oophorectomy before menopause and 46-49 years included 8 age 50-59, 22 age 60-69, 42 age 70-79, and 26 age 80-89 at the time of cognitive evaluation.

[c] Women without bilateral oophorectomy included 313 age 50-59, 442 age 60-69, 790 age 70-79, and 562 age 80-89 at the time of cognitive evaluation.

[d] β coefficients, 95% CIs, and *P* values were calculated using linear regression models adjusted for age at the time of cognitive evaluation (continuous), years of education (≤12 vs. 13-16 vs. >16), and *APOE* genotype (ε4 carrier vs. all others). We compared women with bilateral oophorectomy before menopause and <46 years with women without bilateral oophorectomy. We also compared 51 women with bilateral oophorectomy before menopause and <40 years with women without bilateral oophorectomy, resulting in β of -0.40 (95% CI, -0.66 to -0.15), *P* = 0.002 for global cognition *z* score, β of -0.19 (95% CI, -0.46 to 0.08), *P* = 0.17 for memory *z* score, β of -0.38 (95% CI, -0.65 to -0.11), *P* = 0.005 for attention/executive *z* score, β of -0.30 (95% CI, -0.57 to -0.03), *P* = 0.03 for visuospatial *z* score, β of -0.34 (95% CI, -0.61 to -0.06), *P* = 0.02 for language *z* score, and β of -0.96 (95% CI, -1.71 to -0.21), *P* = 0.01 for Short Test of Mental Status score.

[e] β coefficients, 95% CIs, and P values were calculated using linear regression models adjusted for age at the time of cognitive evaluation (continuous), years of education (≤12 vs. 13-16 vs. >16), and *APOE* genotype (ε4 carrier vs. all others). We compared women with bilateral oophorectomy before menopause and 46-49 years with women without bilateral oophorectomy.

[f] The test was administered face-to-face by a psychometrist, under the supervision of a neuropsychologist (including J.A.F. and M.M.M.). The score range was 0-38.

© 2021 Rocca WA et al. *JAMA Network Open*.
